# 6α-hydroxylated bile acids mediate TGR5 signalling to improve glucose metabolism upon dietary fiber supplementation in mice

**DOI:** 10.1136/gutjnl-2021-326541

**Published:** 2022-06-13

**Authors:** Kassem Makki, Harald Brolin, Natalia Petersen, Marcus Henricsson, Dan Ploug Christensen, Muhammad Tanweer Khan, Annika Wahlström, Per-Olof Bergh, Valentina Tremaroli, Kristina Schoonjans, Hanns-Ulrich Marschall, Fredrik Bäckhed

**Affiliations:** 1 The Wallenberg Laboratory, Department of Molecular and Clinical Medicine, Sahlgrenska Academy, University of Gothenburg, Gothenburg, Sweden; 2 Novo Nordisk Foundation Center for Basic Metabolic Research, Faculty of Health Sciences, University of Copenhagen, Copenhagen, Denmark; 3 ETH Zurich, Zurich, Zürich, Switzerland

**Keywords:** bile acid metabolism, dietary fibre, diabetes mellitus, glucagen-like peptides

## Abstract

**Objective:**

Dietary fibres are essential for maintaining microbial diversity and the gut microbiota can modulate host physiology by metabolising the fibres. Here, we investigated whether the soluble dietary fibre oligofructose improves host metabolism by modulating bacterial transformation of secondary bile acids in mice fed western-style diet.

**Design:**

To assess the impact of dietary fibre supplementation on bile acid transformation by gut bacteria, we fed conventional wild-type and TGR5 knockout mice western-style diet enriched or not with cellulose or oligofructose. In addition, we used germ-free mice and in vitro cultures to evaluate the activity of bacteria to transform bile acids in the caecal content of mice fed with western-style diet enriched with oligofructose. Finally, we treated wild-type and TGR5 knockout mice orally with hyodeoxycholic acid to assess its antidiabetic effects.

**Results:**

We show that oligofructose sustains the production of 6α-hydroxylated bile acids from primary bile acids by gut bacteria when fed western-style diet. Mechanistically, we demonstrated that the effects of oligofructose on 6α-hydroxylated bile acids were microbiota dependent and specifically required functional TGR5 signalling to reduce body weight gain and improve glucose metabolism. Furthermore, we show that the 6α-hydroxylated bile acid hyodeoxycholic acid stimulates TGR5 signalling, in vitro and in vivo, and increases GLP-1R activity to improve host glucose metabolism.

**Conclusion:**

Modulation of the gut microbiota with oligofructose enriches bacteria involved in 6α-hydroxylated bile acid production and leads to TGR5-GLP1R axis activation to improve body weight and metabolism under western-style diet feeding in mice.

**What is already known about this subject?**

- Dietary fibres modulate gut microbiota activity to increase short-chain fatty acid production to affect host body weight and glucose metabolism.
- 6α-hydroxylated bile acids are reduced in patients with type 2 diabetes and possess antidiabetic effects by stimulating TGR5 activity in mice.

**What are the new findings?**

- Oligofructose fibre modulates gut microbiota activity to affect gut bile acid transformation.
- Oligofructose enriches bacterial species involved in production of 6α-hydroxylated bile acids.
- Oligofructose regulates body weight gain and glucose metabolism in a TGR5-dependent manner.

**How might it impact on clinical practice in the foreseeable future?**

- Oligofructose could be a therapeutic tool in patients with type 2 diabetes to enrich bacterial species involved in 6α-hydroxylated bile acids.
- Develop a symbiotic product with oligofructose and bacteria producing 6α-hydroxylated bile acids.

## Introduction

Primary bile acids are steroid compounds that are synthesised from cholesterol in the liver, conjugated either to taurine or glycine and secreted into the small intestine as tauro-cholic or glyco-cholic acid ((T/G)-CA) and tauro-chenodeoxycholic or glyco-chenodeoxycholic acid ((T/G)-CDCA) in humans and tauro-alpha/beta-muricholic acids (Tα/βMCA) in mice. Within the gut, primary bile acids undergo several bacterial enzymatic transformations beginning with deconjugation in the small intestine and subsequent modifications in the colon to generate secondary bile acids such as deoxycholic acid (DCA) and lithocholic acid (LCA) from CA and CDCA in humans, and ωMCA, hyocholic acid (HCA), hyodeoxycholic acid (HDCA) and murideoxycholic acid (MDCA) from αMCA and βMCA in mice.^1^ Bile acids are signalling molecules that modulate the activity of several receptors, notably the G protein-coupled bile acid receptor 1 (GBPAR1 or TGR5) and farnesoid X receptor (FXR) and contribute to the regulation of energy expenditure and glucose metabolism.^2–5^

The modern western lifestyle is associated with reduced fibre intake resulting in reduced bacterial diversity and capacity to metabolise dietary fibres.^6 7^ In addition, recent studies have shown that the western lifestyle is associated with bile acid profile dysregulation, which might contribute to the development of chronic inflammatory diseases such as type 2 diabetes (T2D) and colon cancer^8 9^ and might be alleviated by dietary fibre supplementation.^9 10^ Dietary fibres are essential nutrient sources for specific bacteria and help to maintain gut microbiota function, richness and stability. These complex carbohydrates contribute to host health through production of bacterial metabolites such as short-chain fatty acids (SCFA).^11 12^ Soluble dietary fibres, such as oligofructose (OFS), contribute to the prevention of obesity and T2D development through production of SCFAs.^13 14^ Also, OFS supplementation increases the abundance of specific bacterial genera, for example, *Bifidobacterium*, that may affect production of additional bacterial metabolites, including bile acids.^14^ Although bile acid composition can be affected by diet and in particular by dietary fibres,^15^ it is unclear if OFS contributes to host metabolic improvement under western-style diet challenge by modifying the ability of gut microbiota to transform bile acids.

Here, we address if the soluble dietary fibre OFS regulates host body weight and glucose metabolism by modifying gut bile acid composition. We observed that OFS supplementation affected bacterial bile acid transformation by modifying gut microbiota composition, which reduced body weight gain and glucose metabolism by modulating TGR5.

## Results

### Soluble dietary fibre increases caecal and portal secondary bile acid levels in parallel with improved glucose metabolism

**Figure 1:**
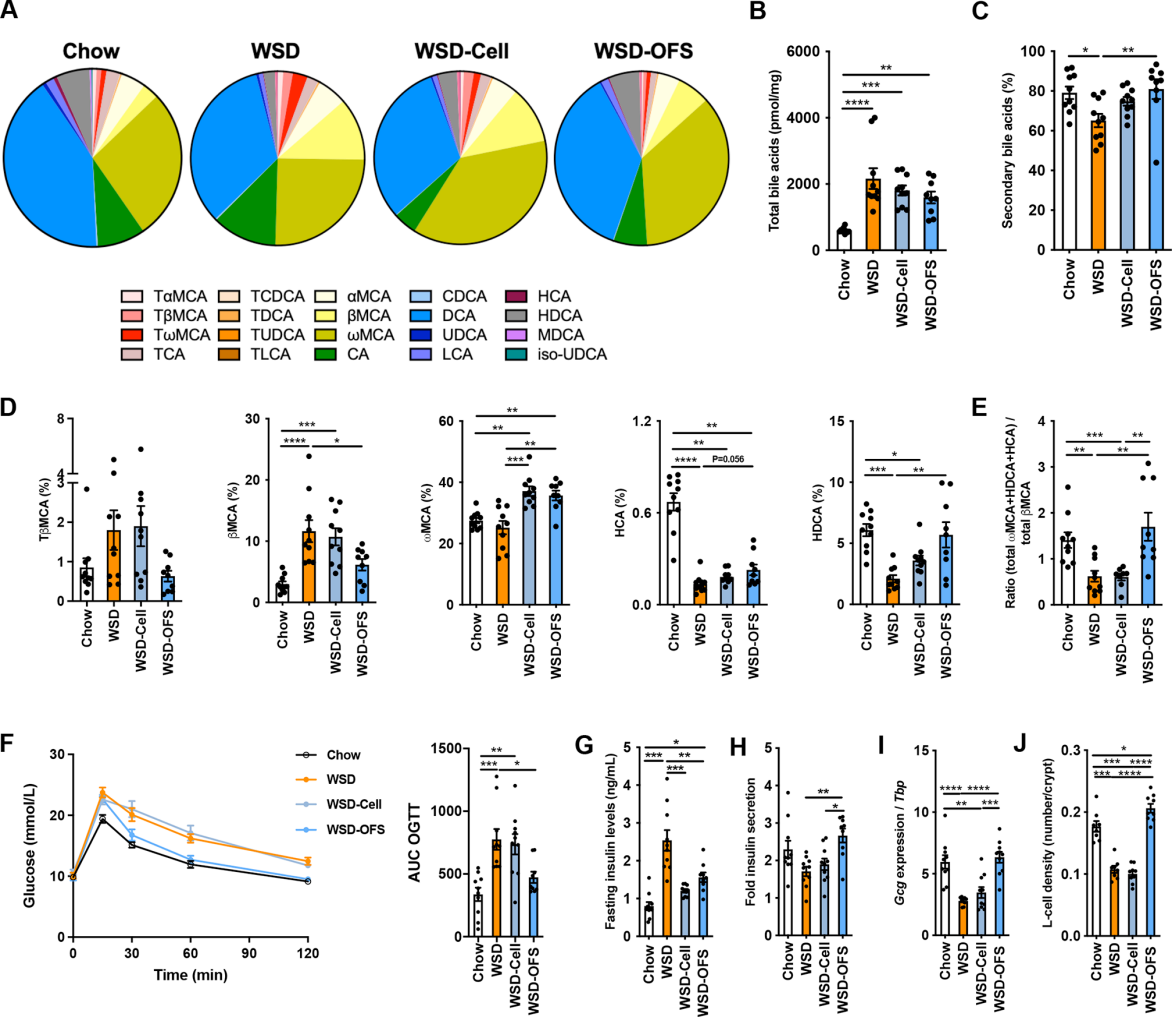
Oligofructose (OFS) supplementation increases caecal 6α-hydroxylated bile acid levels. (A) Bile acid profile in the caecum. (B) Total and secondary bile acid levels in the caecum. (C) Caecal levels of tauro-beta muricholic acid (TβMCA), beta muricholic acid (βMCA), omega muricholic acid (ωMCA), hyocholic acid (HCA) and hyodeoxycholic acid (HDCA) as well as the ratio of conjugated and unconjugated ωMCA, HDCA and HCA over conjugated and unconjugated βMCA levels. (D) Oral glucose tolerance test (OGTT) and its corresponding area under the curve (AUC). (E) Insulin levels, at fasting and 15 min during OGTT. (F) Colonic *Gcg* gene expression and L-cell density. Data are presented as mean±SEM of n= 9−10 per group. Kruskal-Wallis test with multiple comparison test using the original false discovery rate method of Benjamini and Hocheberg was performed for panels B and C. One-way analysis of variance was performed followed with post-hoc Tukey’s test for panels D to F. *p<0.05, **p<0.01, ***p<0.001 and ****p<0.0001 (see also online supplemental figures 1 and 2). DCA, deoxycholic acid; LCA, lithocholic acid; WSD, western style diet.

To investigate if OFS or cellulose affected bile acid metabolism, we fed male C57BL/6J mice chow or western style diet (WSD) or WSD supplemented with 10% cellulose (WSD-Cell) OFS (WSD-OFS) for 8 weeks. Caecal bile acid profile was altered under WSD challenge (figure 1A), and total bile acid levels were higher in mice fed with WSD (alone and when supplemented with dietary fibres) than in chow-fed mice (figure 1B). Total levels of secondary bile acid (BA) in the caecum were reduced in mice fed WSD compared with chow-fed mice, but this reduction was not observed in mice fed WSD-OFS (figure 1C). The increase in secondary bile acid levels in WSD-OFS fed mice compared with WSD group was mainly driven by higher levels of 6α-hydroxylated bile acids, for example, ωMCA, HCA and HDCA, while the levels of precursors TβMCA and βMCA were decreased (figure 1D); LCA and DCA remained unchanged (online supplemental figure 1A). The ratio of ωMCA (TωMCA+ωMCA), HCA and HDCA to βMCA (TβMCA+βMCA) was increased in WSD-OFS-fed mice (figure 1E), suggesting increased microbial bile acid transformation of primary bile acids following OFS supplementation. Similar changes were observed for ωMCA and HDCA levels in the portal vein of WSD-OFS-fed mice (online supplemental figure 1B).

10.1136/gutjnl-2021-326541.supp1 Supplementary data

Gene expression analysis of enzymes implicated in the regulation of classical and alternative pathways for bile acid synthesis showed no differences between WSD-OFS-fed and WSD-fed mice (online supplemental figure 2A). However, *Cyp27a1* and *Cyp2c70* expression were significantly higher in WSD-Cell-fed mice than in chow- or WSD-OFS-fed mice (online supplemental figure 2A). To investigate the effect of dietary fibre supplementation on the expression of key genes in the distal ileum and which are involved in hepatic bile acid regulation, we analysed the expression of *Fgf15*, a factor known to be secreted by the intestinal epithelial cells to modulate bile acid synthesis in the liver. We found that WSD-Cell but not WSD-OFS increased *Fgf15* expression when compared with WSD-fed group (online supplemental figure 2B). Moreover, we did not observe any significant differences in the gene expression of bile acid transporters (*Ibat* and *Ostα*) among the four experimental conditions (online supplemental figure 2B). These results suggest that OFS supplementation but not cellulose promotes microbial conversion of primary to secondary bile acids, without affecting hepatic primary bile acid synthesis.

We also showed that glucose metabolism was impaired in mice fed WSD but not in mice fed WSD-OFS when assessed after 6 weeks with oral glucose tolerance test (OGTT), despite no significant impact of OFS supplementation on body weight gain (figure 1F and online supplemental figure 1C). Mice fed WSD-OFS had lower fasting insulin levels, increased insulin secretion (figure 1G, H) and higher colonic *Gcg* expression and L-cell density, but no differences in the expression of genes involved in L-cell differentiation (*Ngn3*, *NeuroD1* and *Pax6*) compared with WSD-fed mice (figure 1I, J and online supplemental figure 2D). Despite significantly lower body weight gain in mice fed WSD-Cell compared with WSD-fed mice (online supplemental figure 2C), glucose tolerance, insulin secretion and L-cell density of these two groups remained similar (figure 1F–J). Thus, our data suggest that OFS alters bile acid metabolism and improves glucose metabolism, potentially by modulating GLP-1 production.

### Altered gut microbiota composition by OFS supplementation correlates with changes in bile acid composition

**Figure 2:**
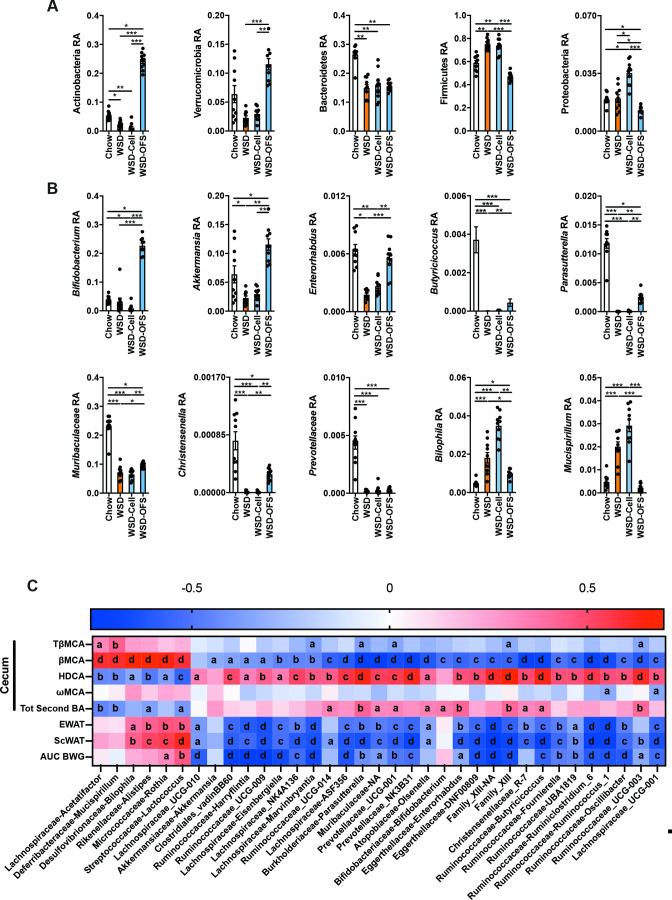
Altered gut microbiota profile by oligofructose (OFS) supplementation correlates significantly with 6α-hydroxylated bile acid levels in the caecum. (A) Relative abundance of bacterial phyla. (B) Relative abundance of selected bacterial families and genera with a false discovery rate of p<0.05. (C) Spearman correlations between the relative abundance of bacterial genera and bile acid profile in the caecum as well as with body weight gain and white adipose tissue weights. Data are presented as mean±SEM. n=9–10 per group. Kruskal-Wallis test was performed followed by a multiple comparison test using the original false discovery rate (FDR) method of Benjamini and Hocheberg. *p<0.05, **p<0.01, ***p<0.001 and ****p<0.0001. For panel C: a: p<0.05, b: p<0.01, c: p<0.001 and d: p<0.0001 (see also online supplemental figures 3 and 4). HDCA, hyodeoxycholic acid; WSD, western style diet; βMCA, beta muricholic acid; ωMCA, omega muricholic acid; TβMCA, tauro-beta muricholic acid,

Supplementation of either cellulose or OFS to WSD resulted in a significant increase in species richness and phylogenetic distance and resulted in different microbiota composition according to Bay-Curtis dissimilarity, unweighted and weighted UniFrac distance analyses (R2: 0.72, p value: 0.0001; R2: 0.63, p value: 0.0001; R2: 0.77, p value: 0.0001; n-perm: 9999). The magnitude of Bray-Curtis dissimilarity and UniFrac distance between the WSD and the WSD-OFS mice were similar to that observed between the WSD and chow mice, and larger than that observed between the WSD and WSD-Cell mice (online supplemental figure 3A–C). Furthermore, OFS supplementation altered gut microbiota composition at the phylum level: the abundance of Actinobacteria and Verrumicrobia increased, driven by increased abundance of *Bifidobacterium* and *Akkermansia* genera, respectively (figure 2A, B). In addition, abundance of *Enterorhabdus*, which belong to the Actinobacteria phylum, increased in mice fed WSD-OFS to levels observed in chow-fed mice. Other genera such as *Parasutterella, Butyricicoccus*, *Muribaculum* and *Christensenella* were higher in WSD-OFS-fed than WSD mice, while *Prevotella* was not increased by OFS supplementation (figure 2B). In addition, *Bilophila,* a genus often associated with metabolic diseases, and *Mucispirillum* decreased in mice fed WSD-OFS to levels observed in chow-fed mice (figure 2B).

To identify bacteria associated with increased ωMCA, HCA and HDCA production, we performed Spearman correlation analyses, which revealed significant correlations (FDR<0.05) between specific bacterial genera and bile acid levels in the caecum (figure 2C). We observed strong positive correlations (ρ>0.5) between the abundance of bacteria belonging to Lachnospiraceae, Prevotellaceae, Muribaculaceae, Burkholderiaceae families as well as Bifidobacteriaceae and Ruminococcaceae with caecal HDCA levels. In addition, the bacteria that correlated negatively with caecal βMCA levels also correlated negatively with body weight gain and adipose tissue weight (figure 2C). In contrast, some bacteria correlated negatively with the abundance of caecal HDCA and positively with caecal βMCA such as *Bilophila*, *Alistipes* and *Mucispirillum* genera. These bacteria correlated positively with adipose tissue weight (figure 2C) and have been described to be associated with glucose dysregulation and metabolic inflammation.^16–18^

### OFS supplementation impacts bacterial activities involved in secondary bile acid biotransformation

To confirm that OFS supplementation directly impacts the production of 6α-hydroxylated bile acids, we cultured the caecal contents from WSD and WSD-OFS mice in a bacterial growth medium (LyBHI) containing primary bile acids obtained from the gallbladder of germ-free (GF) mice. After 24 hours of incubation, the culture media containing either caecal bacteria from the WSD or WSD-OFS-fed mice had lower levels of total primary bile acids and higher levels of total secondary bile acids when compared with time 0 (online supplemental figure 4A). The caecal bacteria from WSD-OFS-fed mice were more efficient at producing secondary bile acids when compared with the caecal content of WSD fed mice, reflecting a higher activity of bile acid transformation (online supplemental figure 4A). ωMCA, HCA and HDCA levels were significantly increased after 24 h in the culture media containing the caecal content of WSD-OFS mice. Also, we observed higher levels of oxidised bile acids such as 3- and 7-oxo-cholic acids, respectively (online supplemental figure 4B), which are intermediates in secondary bile acid synthesis.^19^

**Figure 3:**
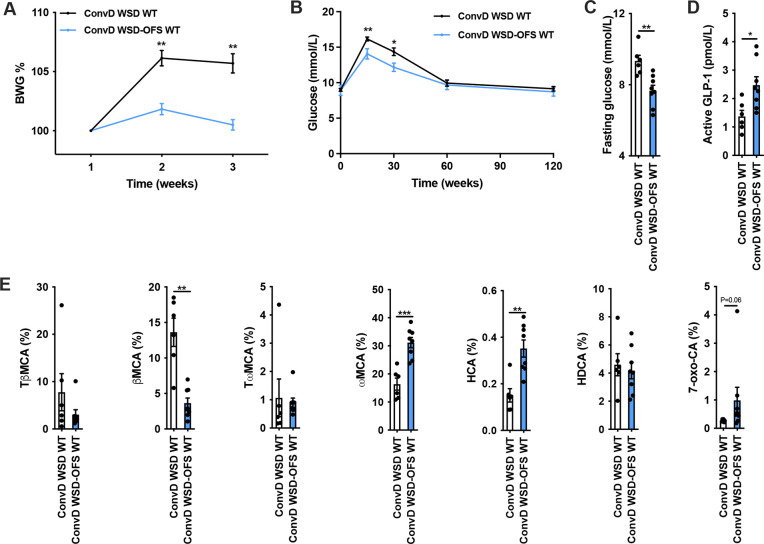
Oligofructose (OFS) supplementation increased the capacity of gut bacteria to produce 6α-hydroxylated bile acids in vivo. (A and B) Body weight gain (BWG) and oral glucose tolerance test (OGTT), respectively of wild-type (WT) germ-free mice colonised with the caecal content of western style diet (ConvD WSD) or western style diet enriched with oligofructose fed mice (ConvD WSD-OFS). (C) Fasting glucose levels before OGTT. (D) Active GLP-1 levels in the portal vein. (E) Caecal bile acid levels. Data are presented as mean±SEM. Two-way analysis of variance with Bonferroni’s multiple comparison test was performed for panels A and B and Mann-Whitney non-parametric test was used when two groups were compared (panels C–E). n=6–8 per group. *p<0.05, **p<0.01 and ***p<0.001 (see also online supplemental figure 5). HCA, hyocholic acid; HDCA, hyodeoxycholic acid; βMCA, beta muricholic acid; ωMCA, omega muricholic acid; TβMCA, tauro-beta muricholic acid.

To investigate if the WSD-OFS-altered gut microbiota improved glucose metabolism in vivo, we colonised wild-type (WT) GF mice fed on chow diet with the caecal content harvested from of WSD (ConvD WSD) or WSD-OFS (ConvD WSD-OFS) mice for 3 weeks. The mice colonised with microbiota from WSD-OFS fed mice had lower body weight gain due to lower accumulation of body fat while the weights of caecal and liver tissues remained unchanged (figure 3A and online supplemental figure 5A). In addition, ConvD WSD-OFS mice displayed reduced fasting glucose levels, increased active GLP-1 levels and improved glucose metabolism (figure 3B–D). Furthermore, ConvD WSD-OFS mice had increased levels of caecal ωMCA and HCA while βMCA was significantly decreased when compared with ConvD WSD mice (figure 3E and online supplemental figure 5B, C). These findings suggest that OFS supplementation enriches bacterial species capable of producing 6α-hydroxylated bile acids potentially from βMCA.

### The beneficial effects of OFS supplementation on body weight and glucose metabolism are dependent on TGR5

**Figure 4:**
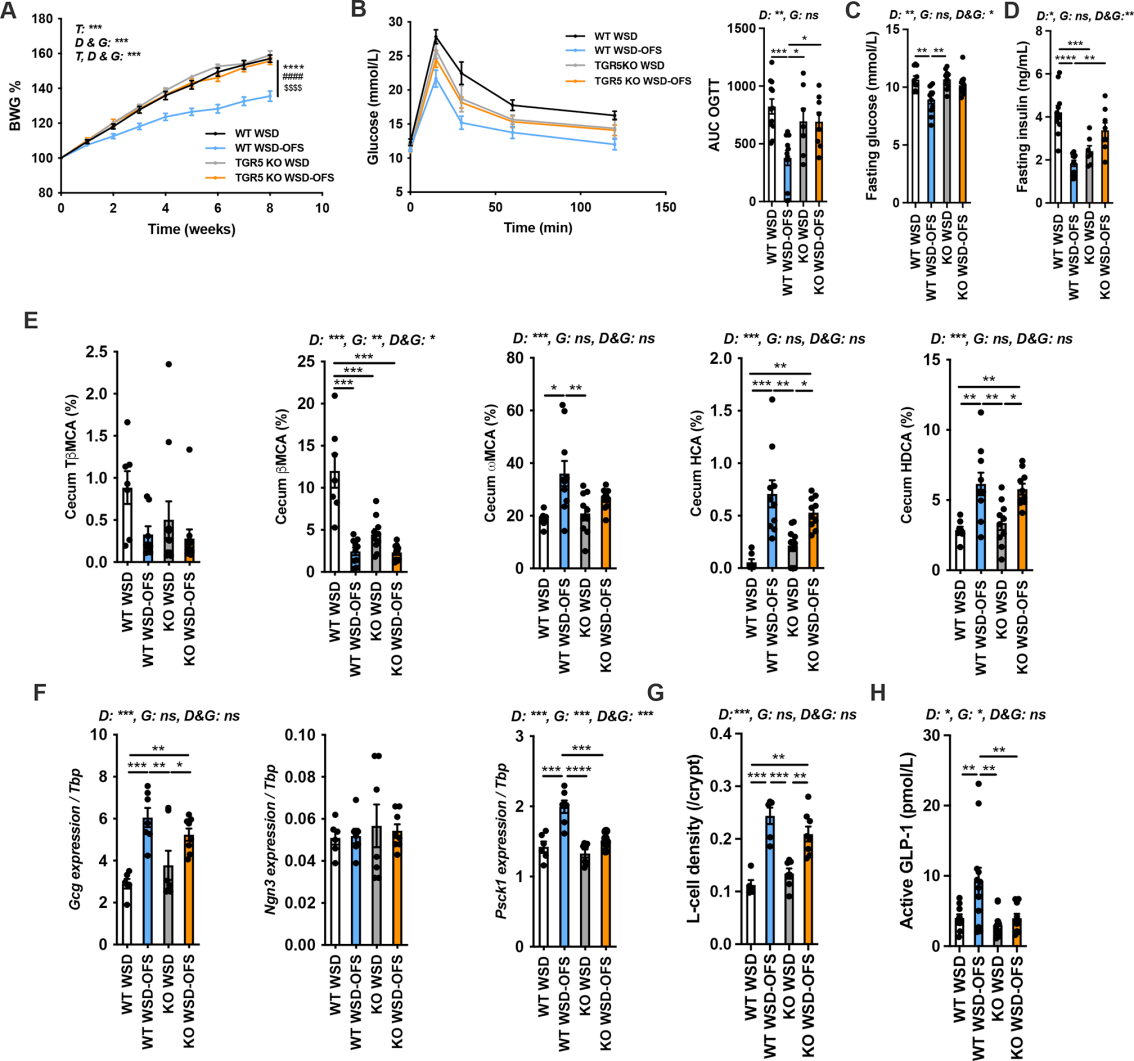
The beneficial metabolic effects of oligofructose (OFS) supplementation are dependent on TGR5 pathway activity. (A and B) Body weight gain (BWG) (n=10–12 per group) and oral glucose tolerance test (OGTT) (n=7–11 per group) of wild-type (WT) and TGR5 KO mice. (C) Fasting glucose levels (n=10–12 per group). (D) Fasting insulin levels (n=7–11 per group). (E) Caecal bile acid levels (n=7–10 per group). (F) Colonic gene expression of *preproglucagon* (*Gcg), neurogenin 3 (Ngn3*) and *proconvertase 1* (*Pcsk1*) (n=6–8 per group). (G) Colonic L-cell density (n=5–8 per group) and active GLP-1 levels in the portal vein (10–12 per group). Data are presented as mean±SEM. Mixed-effects model test was used for BWG analysis (****p<0.0001: WT WSD-OFS vs WT WSD, *####*p<0.0001: WT WSD-OFS vs TGR5KO WSD, $$$$p<0.0001: WT WSD-OFS vs TGR5KO WSD-OFS). Two-way analysis of variance with Bonferroni’s multiple comparison test was performed for panels B to G. D: diet, G: genotype, D&G: diet×genotype. *p<0.05, **p<0.01, ***p<0.001 and ****p<0.0001 (see also online supplemental figure 6). HCA, hyocholic acid; HDCA, hyodeoxycholic acid; βMCA, beta muricholic acid; ωMCA, omega muricholic acid; TβMCA, tauro-beta muricholic acid; WSD, western style diet.

To investigate if the bile acid receptor TGR5 was required for the beneficial effects of OFS, we fed WT and TGR5 KO mice WSD with or without OFS. In WT mice, OFS supplementation significantly reduced body weight gain, adiposity and fasting insulin levels and improved fasting glucose as well as glucose intolerance (figure 4A–D and online supplemental figure 6A). Furthermore, WSD-fed TGR5 KO mice had lower fasting insulin levels than WSD-fed WT mice; however, OFS supplementation in TGR5 KO mice failed to reduce body weight gain or liver weight and prevent adipose tissue expansion (figure 4A–D and online supplemental figure 6A). Also, WSD-OFS feeding did not lower fasting glucose levels or improve glucose tolerance despite higher insulin secretion during OGTT in TGR5 KO mice when compared with TGR5 KO mice on WSD (figure 4A–D and online supplemental figure 6A, B). The levels of caecal ωMCA, HDCA or HCA were comparable between WT and TGR5 KO mice fed with WSD-OFS (figure 4E and online supplemental figure 6C), while the levels of ωMCA and HDCA in portal vein increased only in WT WSD-OFS fed animals and not in WSD-OFS TGR5 KO group when compared with their corresponding controls WT WSD and TGR5 KO WSD, respectively (online supplemental figure 6D). Colonic *Gcg* and *Ngn3* expression and L-cell density were similar between TGR5 KO and WT WSD-OFS-fed mice (figure 4F, G). Expression levels of *Pcsk1*, which encodes the enzyme pro-convertase 1/3 (PC1/3) and plays a key role in GLP-1 release by intestinal L-cells was significantly increased in proximal colon of OFS-treated WT but not TGR5 KO mice fed with the same diet (figure 4F). This was further highlighted by assessing active GLP-1 levels in the portal vein (figure 4G). These results suggest that OFS supplementation promotes the activation of TGR5 and increases GLP-1 secretion potentially through 6α-hydroxylated bile acids.

To investigate if the effect of metabolites produced by the altered microbiota following OFS supplementation also is dependent on TGR5, we next transplanted caecal content from WT mice fed WSD with or without OFS to GF TGR5 KO mice (ConvD WSD and ConvD WSD-OFS, respectively). In contrast, to ConvD WT mice (figure 3), ConvD WSD-OFS TGR5 KO did not show reduced body weight and fasting glucose or increased glucose tolerance and GLP-1 levels despite increased 6α-hydroxylated bile acids in portal vein when compared with ConvD WSD TGR5 KO mice (online supplemental figure 6E–I). Taken together, these results indicate that WSD-OFS induces production of 6α-hydroxylated bile acids, which can mediate metabolic benefits in a TGR5-dependent fashion.

### HDCA activates TGR5 in vitro and in vivo

To investigate if ωMCA or HDCA could function as TGR5 agonists, we evaluated the direct impact of these two bile acids on TGR5 activity using bioluminescence resonance energy transfer technique (BRET). HDCA (10 µM) induced TGR5 signalling with similar amplitude as the positive control (100 nM Merck V) (online supplemental figure 7A). In contrast, ωMCA did not induce TGR5 signalling at 10 µM and we observed a modest activation of TGR5 signalling at 100 µM (online supplemental figure 7A).

**Figure 5:**
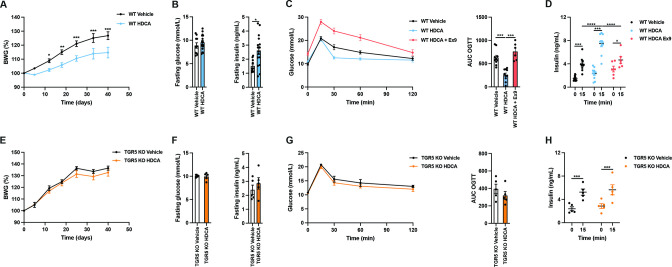
Hyodeoxycholic acid (HDCA) treatment improves host glucose metabolism in a TGR5-dependent mechanism. (A) Body weight gain (BWG) of wild-type (WT) mice treated orally with vehicle or 50 mg/kg of HDCA. (B) Fasting glucose and insulin levels. (C) Oral glucose tolerance test (OGTT) of WT mice treated intraperitoneally with vehicle or 5 µg of Exendin 9–39 20 min before OGTT. (D) Insulin levels during OGTT. (E) BWG of TGR5 KO mice treated orally with vehicle or HDCA (50 mg/kg of body weight). (F) Fasting glucose and insulin levels. (G) OGTT of TGR5 KO mice. (H) Insulin levels during OGTT. Data are presented as mean±SEM. Two-wayanalysis of variance with Bonferroni’s multiple comparison test was performed for BWG analysis and insulin secretion test. Mann-Whitney non-parametric test was used when two groups were compared. n=6–10 per condition for WT group and n=5 per condition for TGR5 KO group. *p<0.05, **p<0.01, ***p<0.001 and ****p<0.0001 (see also online supplemental figure 7).

To investigate if HDCA supplementation is sufficient for improving glucose metabolism in a TGR5-dependent manner, we treated WSD-fed WT and TGR5 KO mice with either vehicle (olive oil) or HDCA (50 mg/kg of BW) by intragastric gavage three times a week for 6 weeks. Body weight gain was reduced, and glucose metabolism was improved by HDCA treatment in WT mice (figure 5A–D and online supplemental figure 7B) but not in TGR5 KO mice (figure 5E–H and online supplemental figure 7B). HDCA supplementation increased fasting insulin without impacting glucose levels in WT mice while no effects have been observed in TGR5 KO mice (figure 5B, F). Furthermore, WT HDCA-treated mice had higher insulin levels after 15 min of glucose challenge compared with the TGR5 KO HDCA-treated mice (figure 5D, H). Additionally, the inhibition of GLP-1R by Exendin 9-39 abolished the improvement in glucose tolerance and insulin secretion after HDCA supplementation in WT mice (figure 5C, D). HDCA treatment did not increase colonic and ileal *Gcg* expression or FXR activity, as reflected by the absence of impact of HDCA on *Fgf15* expression in distal ileum (online supplemental figure 7C, D), or basal active GLP-1 levels in the portal vein after 2 hours of HDCA supplementation (online supplemental figure 7E).

### Exendin-4 supplementation normalises body weight gain and glucose metabolism of TGR5 KO mice fed with OFS

**Figure 6:**
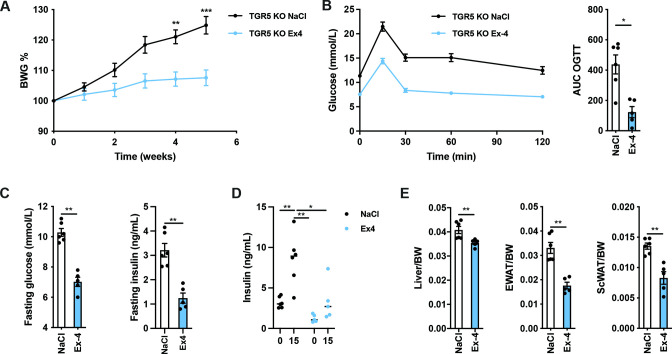
Exendin-4 supplementation improves the metabolic defect of TGR5 KO fed western-style diet (WSD) enriched with oligofructose (OFS). (A and B) Body weight gain (BWG) and oral glucose tolerance test (OGTT) of TGR5 KO mice fed with WSD-OFS and receiving a daily dose of 0.9% of NaCl as a vehicle or exendin-4 (2 nmol/kg/day) using Alzet minipumps. (C) Fasting glucose and insulin levels. (D) Insulin levels during OGTT. (E) Body composition at sacrifice. Data are presented as mean±SEM. Two-way analysis of variance with Bonferroni’s multiple comparison test was performed for BWG analysis and Mann-Whitney non-parametric test was used when two groups were compared. n=5–6 per group. *p<0.05, **p<0.01, and ***p<0.001.

Since intestinal TGR5 activation induces GLP-1 release, we next examined whether GLP-1 acts downstream of TGR5 KO mice fed with WSD-OFS. Thus, we implanted Alzet mini-pumps containing either 0.9% NaCl as vehicle or the stable GLP-1 analogue exendin-4 at 2 nmol/kg/day in TGR5 KO mice fed with OFS-enriched diet for 5 weeks. Exendin-4 administration reduced body weight gain, lowered fasting glucose and insulin levels as well as it improved glucose tolerance (figure 6A–C). However, we did not see higher insulin secretion during OGTT (figure 6D). Treatment of WSD-OFS- fed TGR5 KO mice with exendin-4 reduced liver and adipose tissue weights (figure 6E). These results support the hypothesis that the beneficial effects of OFS on glucose metabolism are mediated by the TGR5-dependent GLP-1 signalling.

## Discussion

Here, we demonstrated that WSD feeding altered 6α-hydroxylated bile acid production by gut bacteria in mice and that OFS supplementation specifically sustained their levels within the intestinal tract and improved glucose metabolism. We further demonstrated that metabolic benefits of OFS feeding were due to altered gut microbiota and identified 6α-hydroxylated bile acids levels, for example, ωMCA, HCA and HDCA, as mediators for the beneficial effects of OFS in a TGR5-dependent fashion. Finally, our data suggest that this pathway involves GLP-1 release and signalling.

We showed that OFS supplementation lowered TβMCA and βMCA and increased the levels of 6α-hydroxylated bile acids, which were associated with the abundance of several bacterial genera enriched by WSD-OFS feeding. Specifically, decreased TβMCA levels following OFS supplementation may be a result of increased abundance of *Bifidobacterium* and their potential to deconjugate bile acids.^20^ ωMCA and HDCA can be produced either from αMCA or βMCA by epimerisation and dehydroxylation processes, while HCA can be produced by epimerisation.^21 22^ The levels of αMCA remained unchanged after OFS supplementation, which suggests that the increase in 6α-hydroxylated bile acids is mainly due to microbial transformation of βMCA, which is reduced following OFS treatment. Bile acid epimerisation and dehydroxylation can be performed by Eggerthellaceae and Lachnospiraceae families, respectively.^21–23^ Our 16S rRNA gene analysis revealed that OFS enriched bacteria belong to Lachnospiraceae and Eggerthellaceae families (eg, genus *Enterorhabdus*), which strongly correlated with caecal HDCA levels. We thus hypothesise that OFS supplementation maintains physiological levels of key bacteria that rely on dietary fibres to maintain their abundance within the gut^7^ and are required for 6α-hydroxylated bile acid production.

Our study showed that WSD-OFS feeding requires a functional TGR5 to improve host glucose metabolism via GLP-1 secretion, which is in agreement with Thomas *et al* who showed that activating TGR5 by bile acids is essential for GLP-1 secretion.^24^ More importantly, increasing *Pcsk1* gene expression and GLP-1 secretion rather than enhancing *Gcg* gene expression seems to play a key role in improving host glucose metabolism under WSD-OFS feeding. The proconvertase enzyme PC1/3, which is encoded by the *Pcsk1* gene is essential for GLP-1 production and can impact its secretion. Our results showed that WSD-OFS feeding increased intestinal *Pcsk1* expression in a TGR5-dependent fashion, an observation that is supported by Marimoto *et al* showing that *Pcsk1* expression is regulated by TGR5 activation and leads to higher GLP-1 production and secretion by L-cells.^25^ Although we observed similar increase in caecal 6α-hydroxylated bile acids between WT and TGR5 KO mice under WSD-OFS-fed diet reflecting conserved bacterial bile acid transformation between both genotypes, it is important to highlight that the structure of the gut microbiota can differ between the two genotypes. Indeed, a recent study have showed that TGR5 KO mice can lead to a modification of gut microbiota composition, which can impact the effects of WSD on TGR5 KO physiology.^26^ Interestingly, we did not observe an increase of HDCA levels in the portal blood of TGR5 KO mice submitted to WSD-OFS diet suggesting that TGR5 might have an impact on the absorption of a subset of bile acids and that HDCA might act at the apical side of colonic cells to activate TGR5. This hypothesis can be supported by a recent study where the authors showed that sulfated bile acids (cholic acid 7-sulfate) which are poorly observed by the gut are able to activate TGR5 apically and lead to increased GLP-1 secretion.^27^

HDCA supplementation to mice fed with WSD improved glucose parameters. The beneficial effects of HDCA have been highlighted in two recent studies. The first demonstrated that HCA, a precursor of HDCA, is negatively correlated with body weight, insulin resistance and HbA1c in humans^28^ and the second showed that HCA and HDCA are decreased in diabetic subjects and their administration to diabetic mice improved glucose metabolism by acting simultaneously on FXR and TGR5 pathways in a GLP-1-dependent manner.^29^ More recently, Petersen *et al* showed that conjugated 6α-hydroxylated bile acids in serum were associated with human metabolic health and gut microbiota, especially to *Clostridia* species.^30^ Specifically, the glyco-conjugated and tauro-conjugated HCA inversely correlated with body weight, adiposity markers and insulin resistance and positively correlated with gut *Clostridia*. However, metagenomic analyses performed in Petersen *et al* study failed to demonstrate a significant correlation between 6α-hydroxylated bile acids and specific clusters of bacterial genes that might be involved in the synthesis. This might be the result of an uncomplete catalogues of functionally annotated bacterial genes. Thus, it is unclear if the production of HCA and HDCA in humans can be performed by specific gut bacterial species and if dietary fibre supplementation enriches the abundance of bacterial genera that might be involved in the production of 6α-hydroxylated bile acids in humans require further investigations.

HDCA administration to WT mice did not modify FXR activity within the gut, measured by using either *Fgf15* expression in the distal ileum or *Gcg* expression in proximal colon as reporters. This discrepancy observed between our and Zheng study might be explained by the differences in the dose and frequency for HDCA supplementation.^29^ HDCA supplementation in Zheng study increased GLP-1 secretion 15 min after oral gavage of the compound, while we did not observe this increase in our model. However, we measured GLP-1 2 hours after HDCA administration, which might suggest that HDCA act on TGR5 in an acute manner to stimulate GLP-1 secretion. Because we showed that inhibiting GLP-1R activity in WT mice receiving HDCA failed to improve glucose tolerance and displayed lower insulin secretion profile when compared with WT mice our data collectively suggest that HDCA improves glucose metabolism through a GLP-1-dependent mechanism. In conclusion, our findings demonstrate that OFS supplementation improves host body weight gain and glucose metabolism by modulating TGR5 and GLP-1 release and potentially occur via production of 6α-hydroxylated bile acids. Nevertheless, the exact mechanism for how GLP-1 produced by colonic L-cells impact host body weight and glucose metabolism remains to be identified. We suggest that several potential mechanisms can be involved, such as the activation of a gut-brain axis via the release of GLP-1 and the maintenance of a healthy and functional enteric nervous system.^31^ Also, a stimulation of TGR5 can impact host physiology through the modulation of intestinal permeability^32 33^ as well as immune cell function by increasing anti-inflammatory cells.^34^ This might limit endotoxemia, which is associated with obesity and metabolic disturbance developments.^35^

However, in the current study we did not investigate the role of SCFA. OFS is a soluble fibre that is well known to be metabolised by gut bacteria and generate SCFA.^13 36^ Accordingly, the beneficial effects of fermentable complex carbohydrates such as inulin on body weight and glucose metabolism have been demonstrated in several studies^14 37 38^ and to be partly mediated by the activation of the SCFA receptor GPR43.^39^ However, inactivation of GPR43 did not abolish GLP-1 secretion or modified L-cell density highlighting that GLP-1 modulation by inulin supplementation might be independent of SCFA^39^ and might rely on other metabolites produced by the gut microbiota such as secondary bile acids.

Our study extends our understanding on how soluble dietary fibres can contribute to host body weight regulation and metabolic improvement by modifying gut ecology and microbial function. We unravel a new mechanism for how the soluble dietary fibre OFS contribute to glucose homeostasis through stimulation of microbial production of 6α-hydroxylated bile acids leading to TGR5 activation and GLP-1 secretion.

## Materials and methods

### Animals and ethics

Male C57BL/6J mice and WT and TGR5 KO^24^ between 8 and 9 weeks old before switched to experimental diets. GF C57BL/6J mice were maintained in flexible film isolators under a strict 12 hours light cycle with unlimited access to water and food. GF status was routinely tested by anaerobic and aerobic culturing of faecal bacteria and by PCR for bacterial 16S rDNA using the primers 27 F and 1492 R (Key Resources Table). All mouse experiments were approved by the Ethics Committee on Animal Care and Use in Gothenburg, Sweden.

### Caecal content preparation and GF colonisation

One to two entire frozen caecal contents were resuspended in 5 mL of brain heart infusion media supplemented with L-cysteine (0.05%), cellobiose (0.1%), maltose (0.1%) inside a COY chamber.

For GF colonisation, WT and TGR5 KO GF C57BL6/J mice were fasted for 4 hours and colonised with caecal contents of WSD or WSD-OFS fed mice and submitted to a chow diet for a period of 3 weeks. One single administration (200 µL per mouse) was used to colonise the animal by intragastric gavage under sterile hood (Isocage system).

### Diets and pharmacological treatments

Mice were fed either chow (5021 LabDiet) or WSD (ENVIGO) for 8 weeks. The diets were purchased from. Diet compositions are presented in online supplemental table 1).

10.1136/gutjnl-2021-326541.supp2 Supplementary data

For exendin-4 delivery, Alzet osmotic mini pumps containing either 0.9% NaCl as a vehicle or exendin-4 (2 nmol/kg/day; HY-1344, MedChemTronica) were used for a period of 6 weeks. The mini pumps were primed for 24 hours before use and were implanted subcutaneously in the dorsal area under anaesthesia.

For exendin 9-39 administration (4017799.0500, Bachem), a dose of 5 µg/kg of body weight was injected intraperitoneally using 0.9% NaCl 20 min before performing the OGTT.

HDCA (H3878, sigma) was resuspended in olive oil and administered by intragastric gavage 3 days a week at the dose of 50 mg/kg of body weight.

### Metabolic phenotyping by an OGTT

Mice were fasted for 5 hours before performing the glucose tolerance test. A glucose load of 2 g/kg of body weight was administrated by intragastric gavage and blood samples were collected from the tail vein at times 0, 15, 30, 60 and 120 min.

### Bile acid quantification in portal blood and caecum samples

Bile acids were analysed using ultra-performance liquid chromatography-tandem mass spectrometry (UPLCMS/MS) according to previous work.^40^ Briefly, 25 µL of portal plasma and approximately 50 mg of caecal tissues were extracted with methanol containing deuterated internal standards. After 10 min of vortex and 10 min of centrifugation at 10 000*g*, the supernatant was evaporated and reconstituted in methanol:water (1:1). The bile acids were separated on a C18 column (1.7µ, 2.1×100 mm; Kinetex, Phenomenex, USA) using water and acetonitrile as mobile phases and detected using MRM in negative mode on a QTRAP 5500 mass spectrometer (Sciex, Concord, Canada). Quantification was made using external standard curves.

### Insulin and GLP-1 level quantifications

Blood insulin and GLP-1 levels were quantified using Ultra-sensitive Insulin (Crystal Chem) and GLP-1 active v2 (Mesoscale) ELISA kits, respectively. For GLP-1 quantification, portal blood was collected at sacrifice using tubes supplemented with aprotinin and DPP-4 inhibitor. The samples were stored at −80° until analysis.

### GLP-1 positive cells staining in proximal colon

Proximal colon tissue was fixed in 4% paraformaldehyde and embedded in paraffin. 5 µm Sections were stained for GLP-1 positive cells using a GLP-1 antibody at 1:500 times dilution (ab22625, Abcam). The sections were incubated overnight at 4° followed by a staining with a secondary antibody anti-Rabbit (Goat anti-Rabbit, A11070, Life technologies) at 1:500 dilution.

### 16S rRNA gene profiling

Total genomic DNA was extracted from 50 mg of caecum using repeated bead beating and the Nucleospin Soil kit. Samples were extracted in SL2 buffer with SX enhancer and sheared with 6 rounds of bead beating at 5.5 m/s for 60 s in a FastPrep−24 Instrument (MP Biomedicals). 20 ng of DNA were added as template in PCR reactions with dual-indexed primers 515F and 806R targeting the V4 region.^41^ PCR reactions were run in duplicate for 25 cycles; duplicates were combined, purified and prepared for sequencing as previously described.^42^ Negative controls were included for each sample and the absence of detectable products was confirmed by gel electrophoresis.

Amplicons were sequenced using a MiSeq instrument (RTA V.1.17.28, bundled with MCS V.2.5; Illumina) and the V2 kit (2 × 250 bp paired-end reads; Illumina). Reads were processed to obtain Zero-radius operational taxonomic units (Zotus) by compiling the sequences into sets of unique reads and performing error-correction using the UNOISE3 algorithm discarding sequences with fewer than four reads. The Zotus were assigned taxonomy using DADA2’s assignTaxonomy (minBoot=50) and assignSpecies, using formatted version of the Silva V.132 database. A phylogenetic tree of the sequences was created with the help of the MAFFT software V.7.407 and the FastTree software V.2.1.10. Analyses were performed on 588 Zotus representing 76% of the Zotus after filtering those contributing with less than 0.002% of the total amount of reads (2 730 655 reads). The graphical representations and statistical analyses of the microbiota were performed using R V.3.5.1 with packages phyloseq V.1.26 and ggplot2 V.3. Faith’s Phylogentic Diversity and richness were calculated using the Picante V.1.7-package (https://github.com/skembel/picante), and pairwise comparisons between diets were performed using Wilcoxon signed rank test. For analysis of beta-diversity, Bray-Curtis dissimilarity, Weighted and Unweighted UniFrac were calculated using the Vegan V.2.5–4 package (https://github.com/vegandevs/vegan) and ordination by Principal Coordinates Analysis. The Adonis test with 9999 permutations was used to assess similarity of composition and was complemented by an Analysis Of SIMilarities (n=9999) using the Vegan package.

### In vitro caecal tissue cultures

Caecal content (1% w/v) from WSD and WSD-OFS fed mice were inoculated in 3 mL of brain heart infusion media supplemented with L-cysteine (0.05%), cellobiose (0.1%), maltose (0.1%) and 0.4% of bile juice collected from gallbladders of GF mice. The in vitro cultures were incubated in anaerobic conditions (5% hydrogen, 10% carbon dioxide and 85% nitrogen) at 37°C for 24 hours. Samples were collected at times 0 and 24 hours for bile acid quantification via UPLC MS/MS as described above.

### COS7-TGR5 BRET essay

COS-7 cells were obtained from the American Type Culture Collection (ATCC) and maintained at 37°C and 10% CO_2_ in DMEM 1885 supplemented with 10% (v/v) fetal calf serum, 2 mM L-glutamine and 100U penicillin and 0.1 mg streptomycin/mL. For assays, cells were plated at 15 000/well in 96-well solid white tissue culture plates (Greiner) 1 day prior to transient co-transfection using the calcium phosphate co-precipitation method. Cells were transfected with a total of 75 ng DNA/well in a ratio of 1:5 receptor: mouse TGR5 construct (NM_174985 from Origene) and Camyel reporter construct (jiang Li, J boil chem, 2007).

Briefly, DNA is mixed with CaCl (2M) and TE-buffer (10 mM Tris-HCl, 1 mM EDTA, pH 7.5), added to 2XHBS (50 mM Hepes, 280 mM NaCl, 1.5 mM NaH_2_PO_4_, pH 7.2) and incubated for 45 min. at room temperature. The mixture and a final concentration of 100 µM Chloroquine (Sigma Aldrich) are added to the cells and left to incubate for 5 hours at 37°C and 10% CO_2_ before changing medium to fresh maintenance medium. BRET-based cAMP assay was performed 2 days after transfection: cells were washed twice with Hank’s balanced salt solution pH 7.4 (HBSS) and incubated in HBSS for 30 min at 37°C (ambient air) prior to BRET measurements. BRET measurements were carried out with a Clariostar plate reader (BMG LabTech). Emission signals from *Renilla* luciferase and YFP were measured simultaneously using a BRET filter set (475-30/535-30). Cells were assayed for 60 min. in a total of 100 µL HBSS containing stated ligands and 5 µM coelenterazine h (Thermo Fischer Scientific) with temperature set at 37°C. Ligands were ωMCA (Hölzel Biotechnology), HDCA and Merck V (a synthetic TGR5 agonist). The BRET signal was calculated as the ratio of the two detected signals from YFP to Renilla Luciferase: BRET ratio=YFP (525 nm)/rLuc (480 nm). cAMP production was calculated as per cent of maximum BRET net signal (ligand–vehicle) obtained with known agonist Merck V.

### Gene expression analysis

Tissues were collected at sacrifice and snap frozen in liquid nitrogen to avoid tissue degradation. Liver, ileum and proximal colon tissues were ground into powders and 30–60 mg of tissues were used for RNA extraction using RNeasy Kit from Qiagen. cDNA synthesis was performed on 1 µg of RNA using High-Capacity cDNA Reverse Transcriptase kit (Applied biosystems). qPCR analyses were performed using the SYBR Green technology (Bio-Rad) and primer sequences are listed in online supplemental table 2.

### Statistical analyses

Statistical analysis was performed using GraphPad Prism V.9. Two-way analysis of variance (ANOVA) with Bonferroni’s multiple comparisons test was performed to evaluate the mean differences between groups having two independent variables. One-way ANOVA with Tukey’s *post hoc* analysis was used for parametric ANOVA between groups, and Mann-Whitney test was used for pairwise comparisons. The Kruskal-Wallis with Benjamini and Hocheberg method was used for non-parametric ANOVA between groups. Data are presented as mean±SEM unless otherwise noted.

## Data Availability

Data are available upon reasonable request. The 16S data for this study have been deposited in the European Nucleotide Archive (ENA) at EMBL-EBI under accession number PRJEB53027 (https://www.ebi.ac.uk/ena/browser/view/PRJEB53027).
