# Supplementary material for: 6α-hydroxylated bile acids mediate TGR5 signalling to improve glucose metabolism upon dietary fiber supplementation in mice
Source: Gut. 2022 Jun 13;72(2):314–24. doi: 10.1136/gutjnl-2021-326541 (PMC9872241; doi:10.1136/gutjnl-2021-326541)
Supplement: Supplementary data [file gutjnl-2021-326541supp001.pdf]

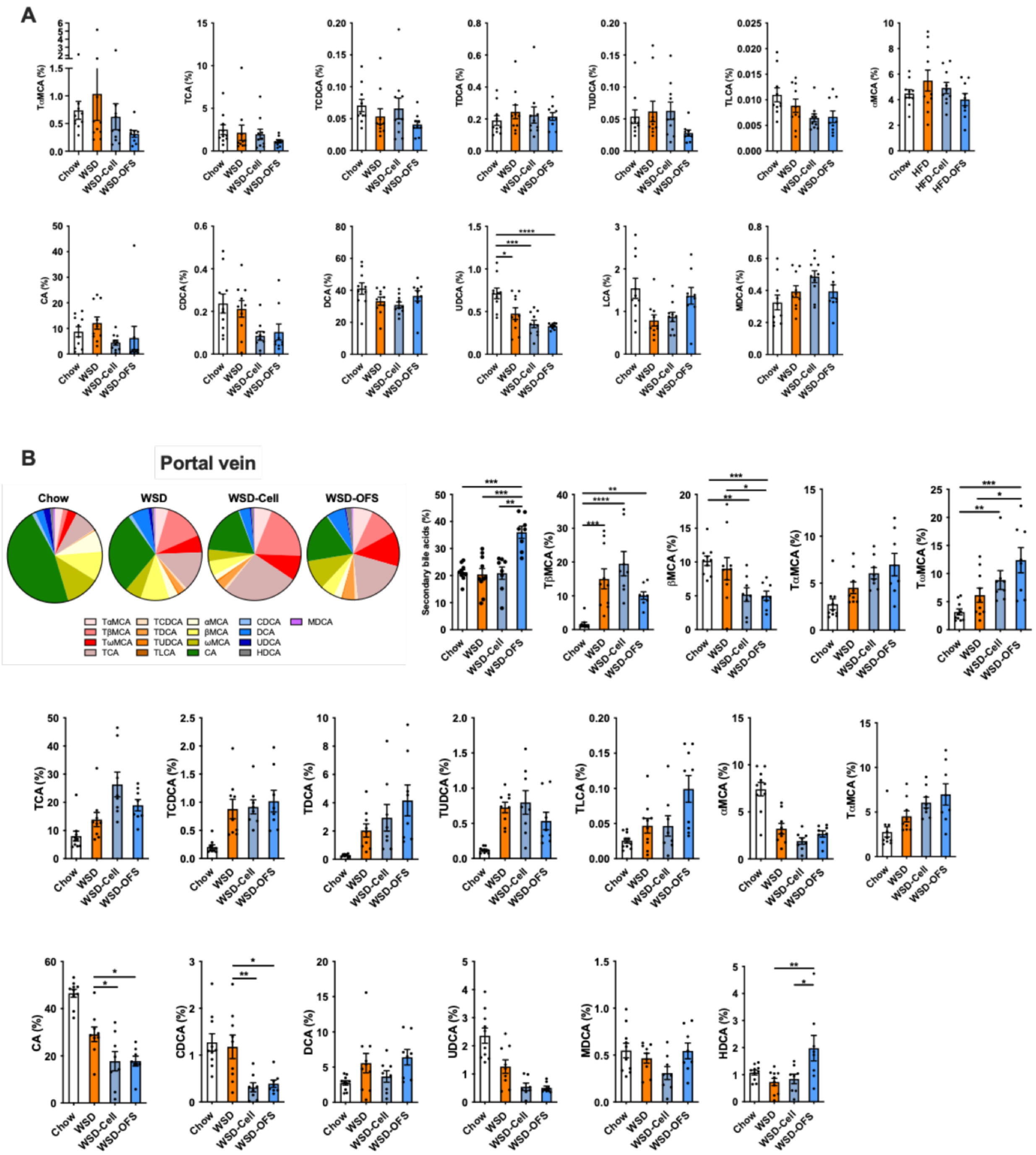

Supplementary figure 1: Soluble dietary fiber impacts cecal and portal bile acid levels

Bile acid profiles in the cecum (A) and porta (B). Data are presented as mean ± S.E.M of n= 8-10 per group. Kruskal-wallis test with multiple comparison test using the original false discovery rate method of Benjamini and Hocheberg was performed. * *p< 0.05*, ** *p< 0.01*, *** *p< 0.001* and **** *p< 0.0001*.

Supplementary figure 1

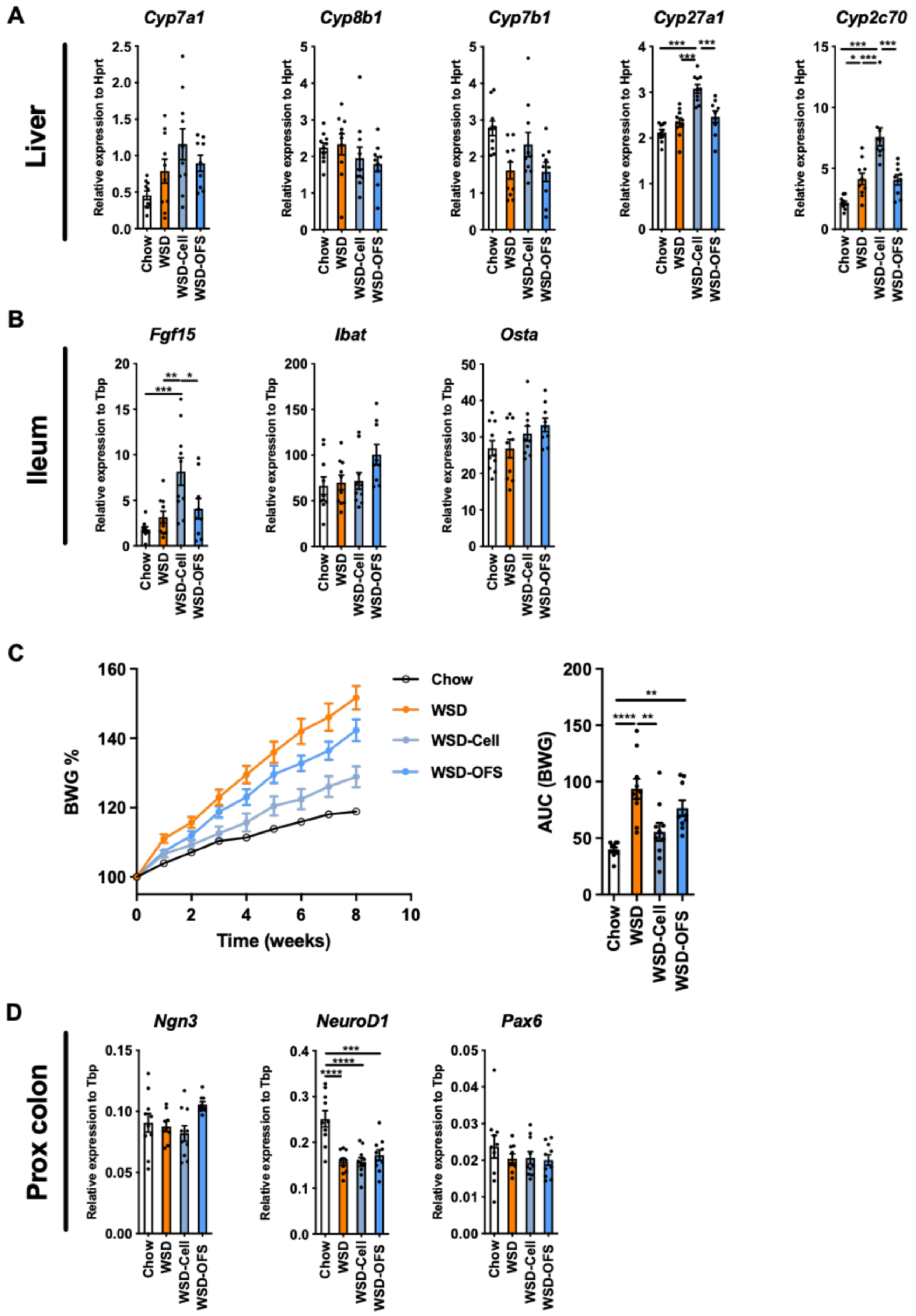

Supplementary figure 2: OFS supplementation alters gene expression profile and promoted lower body weight gain

A. Gene expression analysis of bile acid metabolism genes in the liver. B. Gene expression analysis in the distal ileum. C. Body weight gain and the area under the curve. D. Gene expression analysis in the proximal colon. Data are presented as mean ± S.E.M. n= 9-10 per group. One-way ANOVA was performed followed with post-hoc Tukey's test. * *p< 0.05*, ** *p< 0.01*, *** *p< 0.001* and **** *p< 0.0001.*

Supplementary figure 2

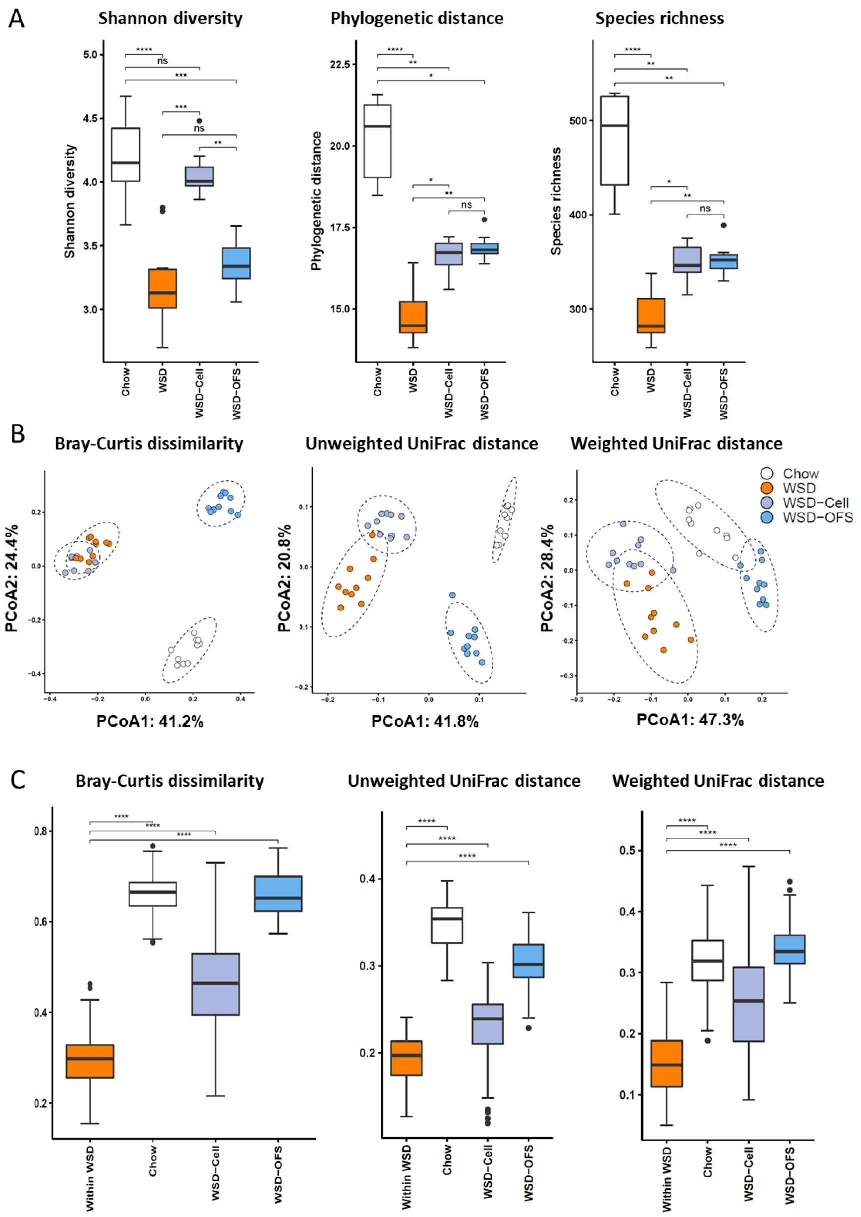

Supplementary figure 3: OFS supplementation alters gut microbiota diversity and richness

A and B. alpha- (Species richness, and Phylogenetic distance, Shannon diversity index) and beta-diversity (Bray-curtis, unweighted- and weighted UniFrac) analyses performed on the cecal microbiota. Alpha diversity was evaluated using Kruskal-wallis test followed by a post hoc Dunn's test, compositional difference in terms of beta-diversity was evaluated using the a permanova test. C. the sample wise dissimilarity and distance within WSD and between WSD and the other diets (Chow, WSD-Cell, and WSD-OFS) evaluated using Wilcoxon's rank sum test. Multiple comparisons were adjusted for false discovery rate using the Benjamini and Hocheberg method. Data are presented as mean ± S.E.M. n= 9-10 per group. ** $p< 0.05$, ** $p< 0.01$, *** $p< 0.001$* and ***** $p< 0.0001$.*

Supplementary figure 3

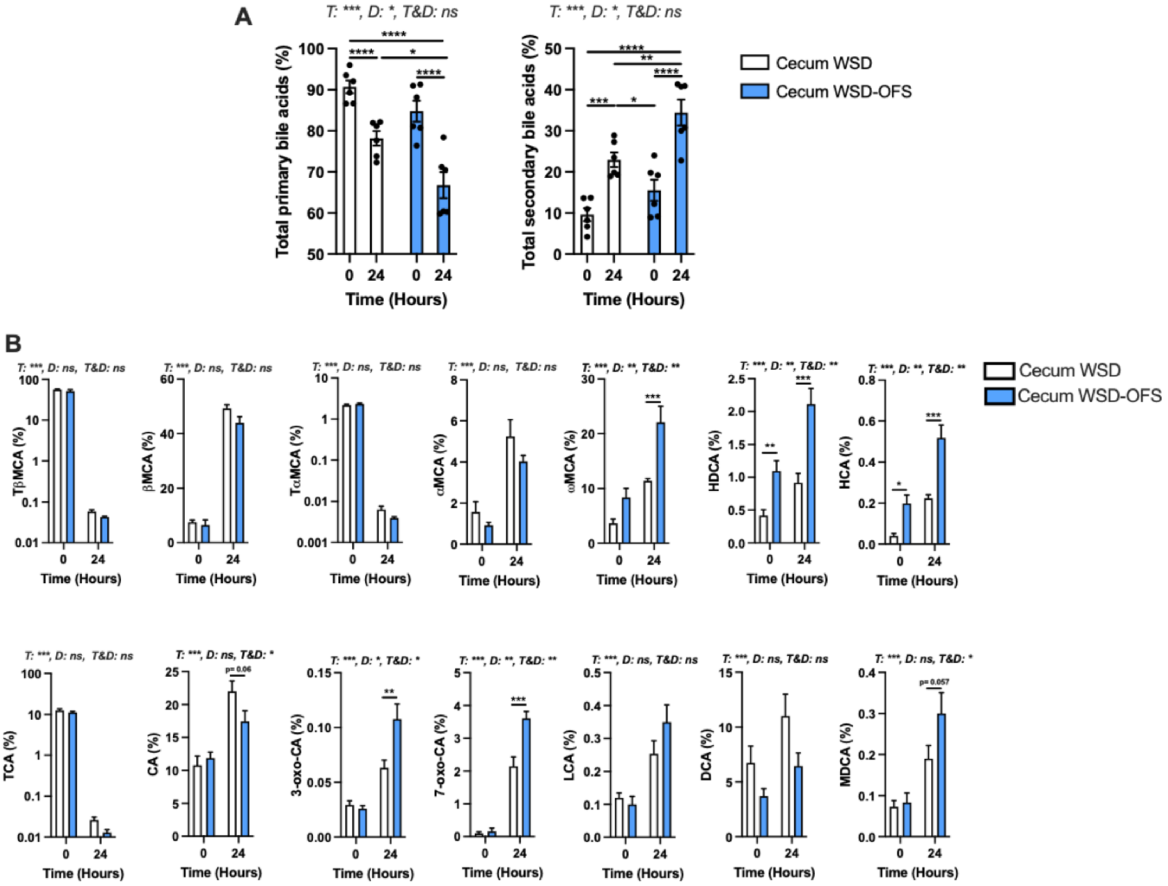

Supplementary figure 4: Cecal bacterial communities of mice fed with WSD-OFS supplementation produce higher secondary bile acids

A. Pie chart representing the average of total primary and secondary bile acids levels *in vitro* at time 0 and 24 hours produced by the cecal bacterial communities of western style diet (WSD) and WSD-OFS fed mice. B. Detailed profile of bile acids produced by the cecal bacterial communities of WSD and WSD-OFS fed mice *in vitro* at time 0 and 24 hours. Data are presented as mean ± S.E.M. n= 6 per group. Two-way ANOVA with Bonferroni's multiple comparison test was performed. T: time, D: diet, T&D: time x genotype. * *p< 0.05, ** p< 0.01, *** p< 0.001, **** p<0.0001.*

Supplementary figure 4

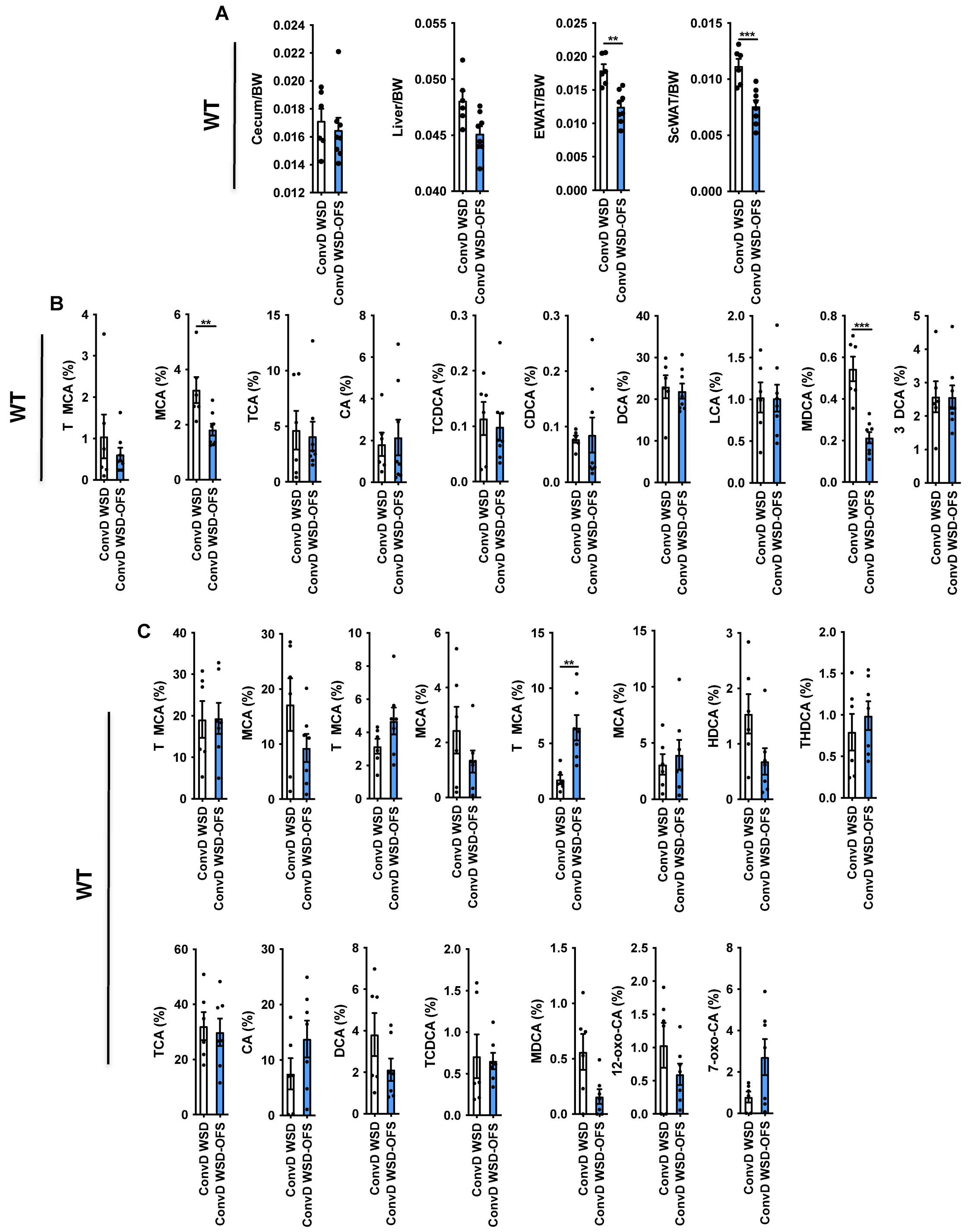

Supplementary figure 5: OFS mediates its beneficial effects on host body composition and bile acid composition through the gut microbiota

A. Body composition of wild-type germ-free (GF) ConvD mice at sacrifice (n= 8 per group). B. Bile acid levels in the cecum. C. Bile acid levels in portal blood (n= 6-8 per group). Data are presented as mean ± S.E.M. Data are presented as mean ± S.E.M. Mann-Whitney non-parametric test. ** *p< 0.01* and *** *p< 0.001.*

Supplementary figure 5

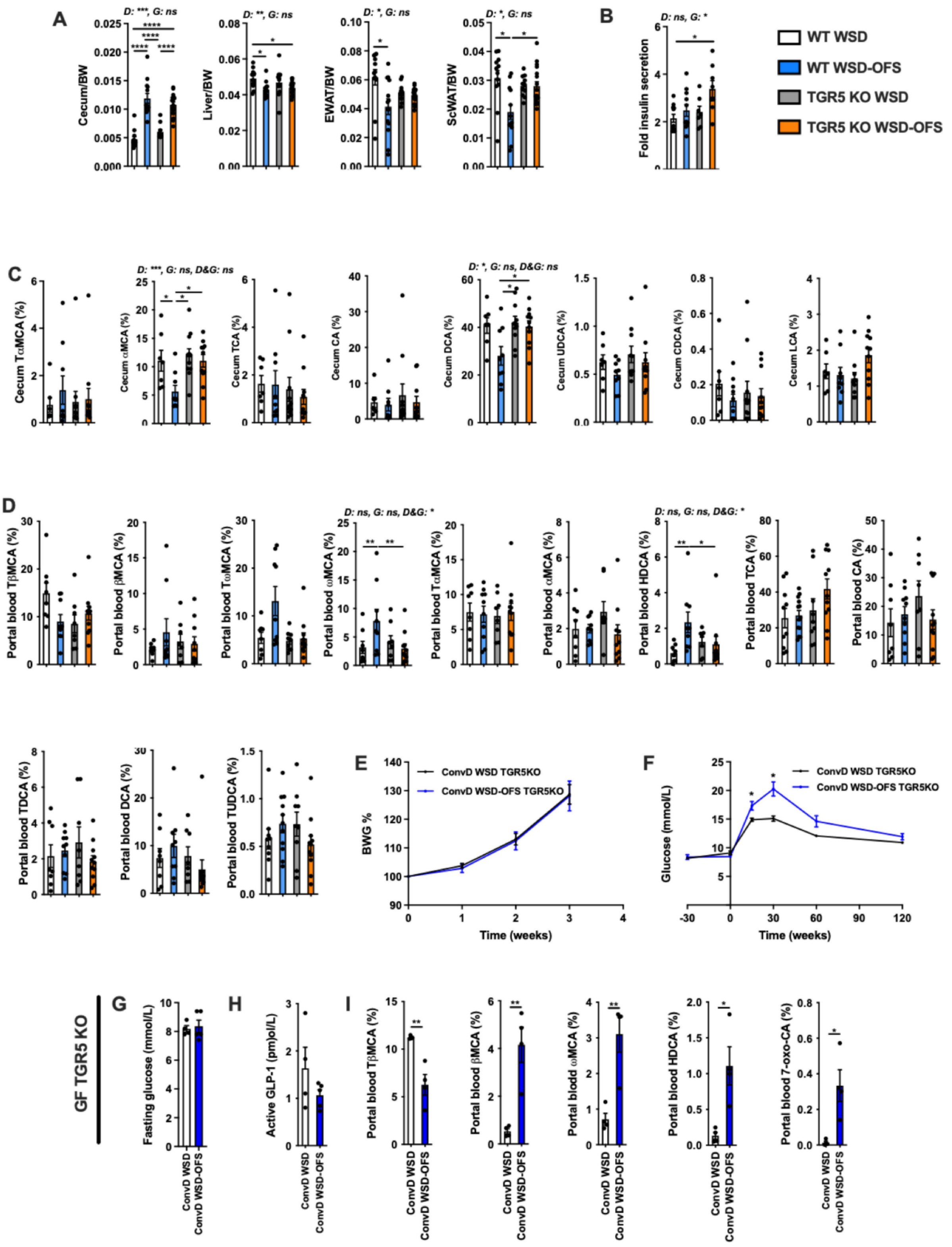

Supplementary figure 6

Supplementary figure 6: The beneficial effects of OFS supplementation on body weight and glucose metabolism are dependent on TGR5

Body composition of wild-type (WT) and TGR5 KO mice at sacrifice (n= 10-12 per group). B. Insulin secretion after 15 min of oral glucose tolerance test (OGTT) (n= 7-11 per group). C and D. Bile acid levels in the cecum and portal blood (n= 7-10 per group). E and F. Body weight gain and oral glucose tolerance test of germ-free TGR5 KO mice colonized with cecal content of WT mice fed with western diet or western diet enriched with oligofructose. G. Fasting glucose levels. H. Active GLP-1 levels in portal vein. I. Bile acid profile in porta. For panels Data are presented as mean ± S.E.M. Two-way ANOVA was performed followed by a multiple comparison test using the original false discovery rate method of Benjamini and Hocheberg. Mann-Whitney non-parametric test was performed when two groups were compared. D: Diet, G: Genotype: D&G: Diet x Genotype. * *p< 0.05*, ** *p< 0.01*, *** *p<0.001*, **** *p<0.0001.*

Supplementary figure 6

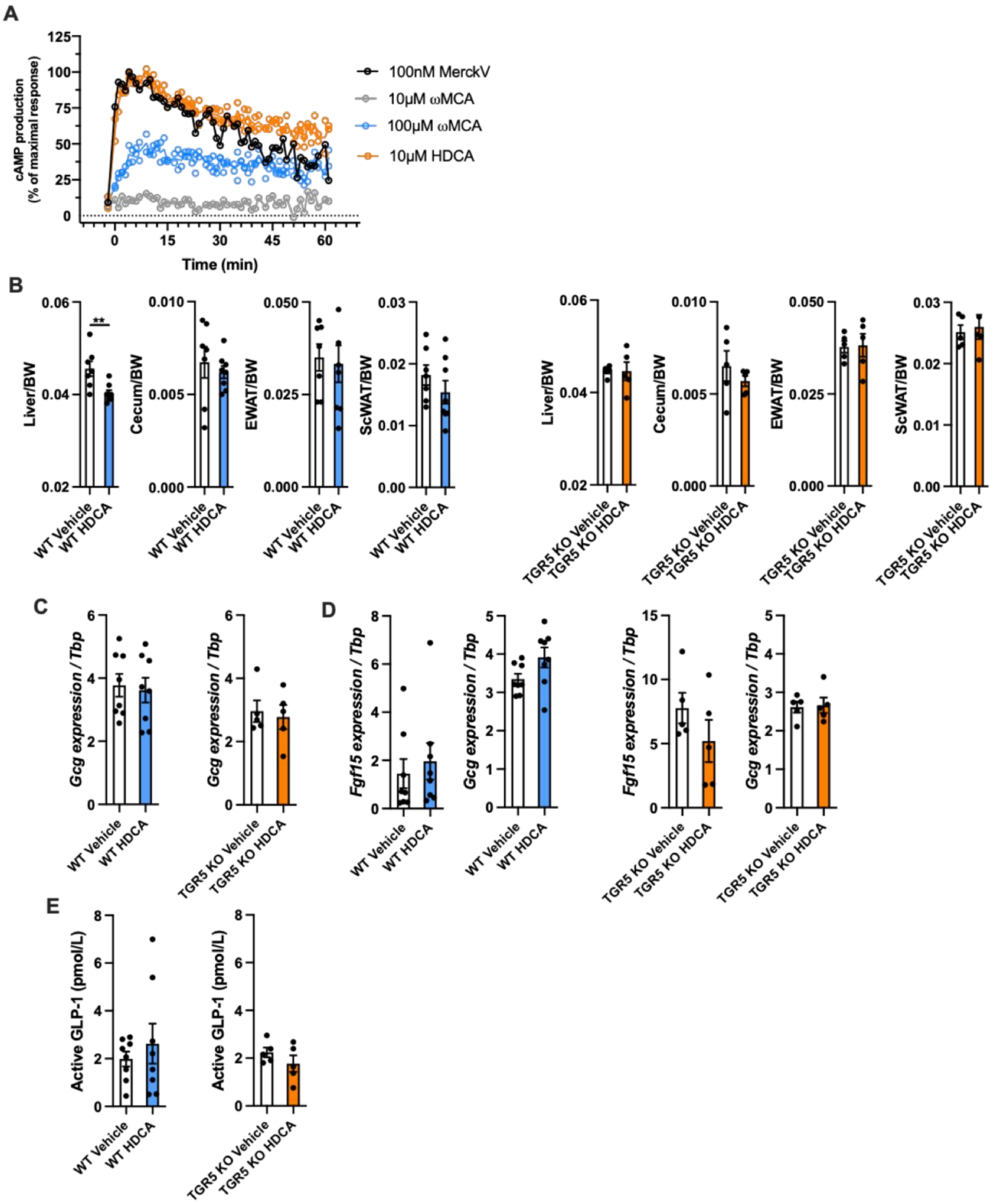

Supplementary figure 7: HDCA activates TGR5 in vitro and requires functional TGR5 in vivo to regulate body composition

A. BRET: cAMP production in Cos7 cells overexpressing TGR5 receptor after treatment with the positive control MERCK V, omega-muricholic acid (ωMCA), or hyodeoxycholic (HDCA). Data are presented as mean ± S.E.M. of 4 independent biological replicates. B. Body composition of wild-type (WT) and TGR5 KO mice at sacrifice treated with vehicle of HDCA (50 mg/kg of BW). C. Colonic gene expression of *Gcg* gene. D. Ileal gene expression of *Gcg* and *Fgf15* genes. E. Active GLP-1 levels in portal vein. Data are presented as mean ± S.E.M. Mann-Whitney non-parametric test was performed. n= 8-10 per condition for WT group and n=5 per condition for TGR5 KO group.

Supplementary figure 7
