# Supplementary material for: 6α-hydroxylated bile acids mediate TGR5 signalling to improve glucose metabolism upon dietary fiber supplementation in mice
Source: Gut. 2022 Jun 13;72(2):314–24. doi: 10.1136/gutjnl-2021-326541 (PMC9872241; doi:10.1136/gutjnl-2021-326541)
Supplement: Supplementary data [file gutjnl-2021-326541supp002.pdf]

Supplementary table 1: Western-style diet composition (ENVIGO)

	**Western Diet No Cellulose – (TD.170882)**	**Western Diet 10% Cellulose – (TD.170883)**	**Western Diet 10% Oligo Inulin – (TD.170885)**
**Formula**		g/Kg	
Casein	236	236	236
DL-Methionine	3.54	3.54	3.54
Sucrose	222.62	182.52	182.52
Corn Starch	160	100	100
Maltodextrin	120	120	120
Cellulose	0	100	0
Oligofructose (Orafti®P95)	0	0	100
Vegetable Shortening, hydrogenated (Primex)	100	100	100
Beef Tallow	100	100	100
Mineral Mix, AIN-93G-MX (94046)	41.3	41.3	41.3
Calcium Phosphate, dibasic	4.72	4.72	4.72
Vitamin Mix, Teklad (40060)	11.8	11.8	11.8
Ethoxyquin, antioxidant	0.02	0.02	0.02
**Selected Nutrient Information**		**% kCal**	
Protein	18	19.6	19.6
Carbohydrate	42.7	37.6	37.6
Fat	39.3	42.8	42.8
**Kcal/g**	**4.6**	**4.3**	**4.3**

Supplementary table 2: Primers used for gene expression analysis

Cyp2c70-F	AGGCTTAGGCATCGTCTTCA
Cyp2c70-R	ATCACATTGCAGGGAACACA
Cyp7a1-F	AGCAACTAAACAACCTGCCAGTACTA
Cyp7a1-R	GTCCGGATATTCAAGGATGCG
Cyp7b1-F	TAGCCCTCTTTCCTCCACTCATA
Cyp7b1-R	GAACCGATCGAACCTAAATTCCT
Cyp8b1-F	GGCTGGCTTCCTGAGCTTATT
Cyp8b1-R	ACTTCCTGAACAGCTCATCGG
Cyp27a1-F	GCCTCACCTATGGGATCTTCA
Cyp27a1-R	TCAAAGCCTGACGCAGATG
Fgf15-F	GAGGACCAAAACGAACGAAATT
Fgf15-R	ACGTCCTTGATGGCAATCG
Fxr-F	TCCAGGGTTTCAGACACT
Fxr-R	GCCGAACGAAGAAACATGG
Gcg-F	GCGAGGGACCTGCCTATGCAAC
Gcg-R	AATGGCGACTTCTTCTGGGAA
Ibat-F	ACCACTTGCTCCACACTGCTT
Ibat-R	CGTTCCTGAGTCAACCCACAT
NeuroD1-F	CCAGGGTTATGAGATCGTC
NeuroD1-R	CGCTCTCGCTGTATGATTT
Ngn3-F	GCATGCACAACCTCAACTC
Ngn3-R	TTTGTAAGTTTGGCGTCATC
Pax6-F	AACAACCTGCCTATGCAAC
Pax6-R	ACTTGGACGGGAACTGACA
Pcsk1-F	CTTTGCTCTGGCCTTGGA
Pcsk1-R	AATCGGCTGTTCACCATCAA
Slc51a (Ostα)-F	TGTTCCAGGTGCTTGTCATCC
Slc51a (Ostα)-R	CCACTGTTAGCCAAGATGGAGAA
